# Genome-Wide Association Analysis of Anoxia Tolerance in *Drosophila melanogaster*

**DOI:** 10.1534/g3.119.400421

**Published:** 2019-07-16

**Authors:** Jacob B. Campbell, Paula F. Overby, Alyx E. Gray, Hunter C. Smith, Jon F. Harrison

**Affiliations:** School of Life Sciences, Arizona State University, Tempe, AZ 85287

**Keywords:** GWAS, DGRP, *D. melanogaster*, hypoxia, stress tolerance

## Abstract

As the genetic bases to variation in anoxia tolerance are poorly understood, we used the *Drosophila* Genetics Reference Panel (DGRP) to conduct a genome-wide association study (GWAS) of anoxia tolerance in adult and larval *Drosophila melanogaster*. Survival ranged from 0–100% in adults exposed to 6 h of anoxia and from 20–98% for larvae exposed to 1 h of anoxia. Anoxia tolerance had a broad-sense heritability of 0.552 in adults and 0.433 in larvae. Larval and adult phenotypes were weakly correlated but the anoxia tolerance of adult males and females were strongly correlated. The GWA identified 180 SNPs in adults and 32 SNPs in larvae associated with anoxia tolerance. Gene ontology enrichment analysis indicated that many of the 119 polymorphic genes associated with adult anoxia-tolerance were associated with ionic transport or immune function. In contrast, the 22 polymorphic genes associated with larval anoxia-tolerance were mostly associated with regulation of transcription and DNA replication. RNAi of mapped genes generally supported the hypothesis that disruption of these genes reduces anoxia tolerance. For two ion transport genes, we tested predicted directional and sex-specific effects of SNP alleles on adult anoxia tolerance and found strong support in one case but not the other. Correlating our phenotype to prior DGRP studies suggests that genes affecting anoxia tolerance also influence stress-resistance, immune function and ionic balance. Overall, our results provide evidence for multiple new potential genetic influences on anoxia tolerance and provide additional support for important roles of ion balance and immune processes in determining variation in anoxia tolerance.

Oxygen limitation plays a key role in many pathologies, some as a result of high tissue metabolism (*i.e.*, cancer) and others from deprivation of O_2_ supply including stroke, heart attack, and sleep apnea (Eltzschig and Eckle 2011). However, as yet, the basic mechanisms by which tissue anoxia and hypoxia negatively impact organisms remain largely elusive. In nature, animals occasionally run the risk of being exposed to low oxygen environments and must respond in a manner to allow them to survive until oxygen levels are restored. Some lower vertebrates (*i.e.*, carp and turtles) and many invertebrates can survive long bouts of anoxia (see reviews in Bickler and Buck 2007; Harrison 2015). In contrast, within mammals, humans can tolerate only a few minutes of anoxia before severe brain damage occurs, while the most tolerant mammal measured to date (naked mole rat) can survive 18 min of anoxic exposure (Park *et al.* 2017). While several studies have examined the variation in anoxia tolerance in various species, we have little understanding of the genetic basis to intraspecific variation in anoxia-tolerance.

The predominant theory, at least for vertebrates and highly-anoxia-tolerant marine invertebrates, is that variation in anoxia tolerance relates to the capacity to maintain ATP levels by matching ATP supply to demand during anoxia (Hochachka *et al.* 1996; Larade and Storey 2002). Variation among vertebrate species in anoxia tolerance has been primarily demonstrated to involve differences in abilities to downregulate energy turnover and/or to upregulate anaerobic ATP production (Hochachka *et al.* 1996). When exposed to anoxia, animals must cope with reduced ATP production and as ATP becomes depleted, ion homeostasis can be disrupted, eventually leading to cell death (Storey and Storey 2007; Galli and Richards 2014). Matching ATP demand and supply allows some anoxia-tolerant species to maintain ATP levels during anoxia, preventing deleterious effects such as decreased pH, altered calcium homeostasis, increased intracellular osmotic pressure, and/or mitochondrial damage (see review in Hochachka and Somero 2002). While many these conclusions were made using interspecific comparisons, this trend may also be true for intraspecific variation. If so, this would suggest that genetic variation in the capacities to down-regulate metabolically demanding activities (*e.g.*, protein synthesis, ion channel activity), or variation in anaerobic capacities might underlie intraspecific variation in anoxia tolerance.

An alternative or complementary view of anoxia tolerance, more supported by studies with insects, is that variation in anoxia tolerance is related to differences in abilities to prevent or repair damage despite loss of most ATP. Insects deplete most of their ATP and ion gradients relatively quickly in anoxia, yet survive for many hours or even days (Wegener 1993; Krishnan *et al.* 1997; Campbell *et al.* 2018; Ravn *et al.* 2019). Previous studies have indicated that mechanisms that protect against protein unfolding can be important in anoxia-tolerance, including induction of heat shock proteins (Azad *et al.* 2011; Deng *et al.* 2018), and trehalose (Chen *et al.* 2002), suggesting that genes involved in coping with protein unfolding might be important in intraspecific variation in anoxia tolerance. Because anoxic exposure is also associated with oxidative damage during anoxia (López-Martínez and Hahn 2012), after reperfusion (Granger and Kvietys 2015) and inflammation (Eltzschig and Carmeliet 2011), genes that affect ROS production, removal, or damage repair, or genes that influence the inflammatory response might be important in mediating intraspecific variation in anoxia tolerance. Because insects deplete ATP and ion gradients during anoxia like mammals, yet survive much longer, they may represent a particularly fruitful group to uncover potential approaches to ameliorate hypoxic-associated damage in human disease.

One strategy for understanding the genetic basis of tolerance to hypoxia is through the use of model organisms and their translational power to understand oxygen-mediated pathways in human disease (Farahani and Haddad 2003). *Drosophila melanogaster* is a well-suited model for multiple reasons—it is hypoxia-tolerant, the majority of major metabolic pathways are conserved between flies and humans, and >60% of the fly genome is conserved humans. While anoxia tolerance of larval *Drosophila* has yet to be thoroughly investigated, there has been extensive research on anoxia tolerance in adult *Drosophila*. Gene expression studies have shown changes in genes involving metabolism, immune response, oxidative stress, and the unfolded protein response in flies exposed to severe hypoxia (Gleadle and Ratcliffe 1998; Liu *et al.* 2006; Zhou *et al.* 2007; Azad *et al.* 2009). In flies exposed to severe hypoxia, an upregulation of Hsp expression corresponds with an increased tolerance (Azad *et al.* 2009), and overexpression of trehalose-6-phosphate synthase (*tps1*) increased survival in anoxia (Chen *et al.* 2003). *dADAR*, also known as *hypnos-2*, is another gene implicated in anoxia tolerance of *D. melanogaster* that was discovered in early experiments investigating the genetic basis of anoxia tolerance (Haddad *et al.* 1997); *dADAR* mutant flies are senstive to anoxia (Ma *et al.* 2001). *dADAR* is involved in RNA editing of ion channels and plays a role in nervous system function (Palladino *et al.* 2000), and additionally been shown to aid in mediating ROS scavenging (Chen *et al.* 2004). Despite these findings, variation in anoxia tolerance at the population level has yet to be examined and to what extent genetic variation affects intra-individual differences in anoxia tolerance in natural populations is still unknown.

Here, we use the *Drosophila* Genetic Reference Panel (DGRP) to conduct a genome-wide association study to identify genetic variation and target genes related to anoxia tolerance in adult and larval *D. melanogaster*. The DGRP consists of 205 fully sequenced inbred strains derived from a single outbred population of flies developed as a resource for whole genome association mapping (Mackay *et al.* 2012). The DGRP is a powerful tool for that can be used for mapping a phenotypic trait to genetic variation. In addition to the GWA, we assessed the extent of genetic correlation between our anoxia tolerance phenotype and other phenotypes measured in the DGRP. Lastly, using a subset of genes and SNPs identified by the GWA, we used an RNAi-based and a SNP-based method to functionally test effects of these genes on anoxia tolerance.

## Methods

### Fly stocks and culture

DGRP lines and RNAi lines under UAS control from the Transgenic RNAi Project (TRiP) (Perkins *et al.* 2015) were obtained from the Bloomington Stock Center. Also acquired from Bloomington, the TRiP progenitor stock lines (BL#36303 & BL#36304) were used for control lines in adult experiments and a ubiquitous GAL4 driver (alphaTub84B; BL#5138) was used as the GAL4 driver for all functional experiments. All flies were maintained on a standard cornmeal molasses diet and reared at 25°.

### Anoxia survival phenotypes

Adults 3-4 days old and late 3^rd^ instar larvae (LL3; identified as individuals lacking food in the gut and climbing out of the media and up the walls of the vials) were collected and separated into groups of 10-20 individuals per replicate, with a minimum of three replicate vials per line. Adults were CO_2_-anesthetized for sex-segregation, and were allowed at least one day to recover before anoxic treatments. Anoxia treatments were conducted as previously described (Campbell *et al.*, 2018); briefly, standard *Drosophila* vials containing food were placed into an air-tight chamber and perfused with humid nitrogen. To ensure the nitrogen was >80% saturated, nitrogen was bubbled through distilled water before entering the chamber. Behavioral responses were rapid—demonstrated by larvae climbing out of the media and adults becoming paralyzed in less than one minute—indicating that induction of anoxia was rapid. We used different durations of anoxic exposure for adults (6 h) and larvae (1 h) to achieve similar, moderate mortality (Callier *et al.* 2015; Campbell *et al.* 2018). After exposure to anoxia, vials were removed from the chamber and placed on their sides in an incubator to allow for recovery under normal conditions; additionally, for larvae, we counted the number of larvae that had climbed out of the media by the end of the 1 h anoxic exposure. After 24 hr, the number of survivors were counted for each vial; adult survivors were counted as the number with the ability to move. Previous data (Callier *et al.*, 2015) show that LL3 pupate within 24 hr after anoxia, and therefore the number of pupae present after 24 hr of recovery was considered the number of surviving larvae. The survival phenotype was presented as the proportion surviving for each replicate.

### Quantitative genetic analyses

A linear mixed model was used to determine variance components in anoxia survival across DGRP lines and between sexes for adults. Variances were portioned for each phenotype using the model: Y= μ+Sex+Line+Sex∗Line+ε, where *Sex* is fixed, *Line* is random, and ε is the error variance. Reduced models were also fit using: Y= μ+Line+ε for each sex separately and for larvae. Variance components were estimated using the restricted maximum likelihood (REML) method. A minimum of three replicates and ten flies per replicate were analyzed per DGRP line and sex, which allowed the calculations of broad-sense heritability (*H^2^*) for each of five analyses: adults pooled by sex, male and female analyzed separately, larvae, and between stages (larvae and adults pooled by sex). Broad sense heritability (*H^2^*) was calculated as $H^{2}=(\sigma_{L}^{2}+\sigma_{SL}^{2})/(\sigma_{L}^{2}+\sigma_{SL}^{2}+\sigma_{E}^{2})$, where $\sigma_{L}^{2}$ is the variance component among lines, $\sigma_{SL}^{2}$ is the line-by-sex or line-by-stage variance component, and $\sigma_{E}^{2}$ is the sum of all other sources of variation. *H^2^* was calculated for larvae and each adult sex separately using $H^{2}=\sigma_{L}^{2}/(\sigma_{L}^{2}+\sigma_{E}^{2})$. We calculated genetic correlations across sexes and life stages to test whether similar genes underlie anoxia tolerance across sexes and life stages, which will influence whether the sexes and life stages might evolve anoxia tolerance independently. Cross-sex and cross-stage genetic correlation (*r_gs_*) was calculated as $r_{gs}=\sigma_{L}^{2}/\surd (\sigma_{LM}^{2}\times \sigma_{LF}^{2})$ for adults and $r_{gs}=\sigma_{L}^{2}/\surd (\sigma_{LL}^{2}\times \sigma_{LA}^{2})$ for life stage, where $\sigma_{L}^{2}$ is the variance component among lines for sexes or life stages combined, $\sigma_{LM}^{2}$ and $\sigma_{LF}^{2}$ are variance components among lines for males and females, $\sigma_{LL}^{2}$ is the variance for component for larvae, and $\sigma_{LA}^{2}$ is the variance component for adults pooled by sex. The calculation of *r_gs_* is an estimate of the genetic basis to a trait that generally falls between 0 and 1; for example, an *r_gs_* value of 1 between adult male and female anoxia tolerance would indicate a near identical genetic architecture while an *r_gs_* of 0 would indicate a sex-specific genetic base to anoxia tolerance with little overlap in genetic architecture.

### Genome-wide association analyses for anoxia tolerance

Associations for anoxia survival were computed using line means for survival phenotypes using the DGRP pipeline (dgrp2.gnets.ncsu.edu); the DGRP pipeline consists of 1,920,276 SNPs with minor allele frequencies (MAF) of 0.05 or greater, and adjusts means for the effects of *Wolbachia* infection and 5 major chromosomal inversions (Huang *et al.* 2014). SNPs significantly associated with anoxia tolerance were identified at a nominal P-value threshold of *P* < 10^−5^. Next, gene ontology analyses were performed using the DAVID Functional Annotation Tool and GOFinder (Huang *et al.* 2009b; a), with Bonferroni correction for multiple tests. All stats were conducted using R software and various packages [doBy (Højsgaard and Halekoh 2016), ggplot2 (Wickham 2009), dplyr (Wickham *et al.* 2017), nlme (Pinheiro *et al.* 2017)].

### Functional experiments

We used UAS-RNAi knockdown lines from TRiP to functionally test candidate genes discovered in the GWA (for adult functional experiments: *babos*, *CG10089*, *MED6*, *PMCA*, *ppk19*, *ppk30*, *SLO2*; and for larval functional experiments: *CG2258*, *CG43795*, *chif*, *Eip63F-1*, *IRSp53*, *Lasp*, *mamo*, *Pde11*, *Prm*, *X11Lβ*). For adult experiments, the GAL4 driver was crossed with virgin females from the UAS-RNAi lines to achieve ubiquitous gene knockdown while the GAL4 driver crossed with the progenitor lines served as controls. We assessed the proportion surviving 6 h of anoxia using the similar methods as stated above; the only difference was that individuals were also grouped by genotyped before exposure. A likelihood ratio test was used to compare RNAi knockdown genotypes to controls for each sex separately. For larvae, similar methods were used as stated above with one essential difference. Because individual larvae could not be genotyped until pupation, survival was counted as the number pupating for each genotype. The *UAS-GAL4* mating scheme we employed produces a ratio of 1:1 (RNAi knockdown:control offspring) for the F_1_ generation; therefore, we used a likelihood ratio test to test for differences in the expected ratio between the two genotypes after 1 h of anoxic exposure to determine if the RNAi knockdown individuals were less likely to survive anoxic exposure.

A second set of experiments were conducted to examine the effects of SNP alleles on anoxia tolerance in adults using methods previously described (Garlapow *et al.* 2015). We selected two significant SNPs (*2L*_9399117_SNP, *2R*_18108684_SNP) identified by the GWAS to assess the effect of SNP alleles on anoxia tolerance in adults. We randomly selected 10 lines from each of the major or minor allele containing DGRP lines. Within each subset of 10, we selected 5 to be the male parent and 5 to be the female parent. This produced 10 F_1_ genotypes that were homozygous for either the major or minor allele while allowing the remaining genetic background to be somewhat outbred. We then assessed the proportion surviving a 6 h exposure to anoxia by counting the number surviving 24 h after exposure. We used a likelihood a ratio test to test for differences between the reference and alternate allele.

### Data availability

Raw phenotypic data for each line can be found in the supplemental material, File S1. Supplemental material available at FigShare: https://doi.org/10.25387/g3.8316494.

## Results

### Quantitative genetics of anoxia tolerance

**Figure 1:**
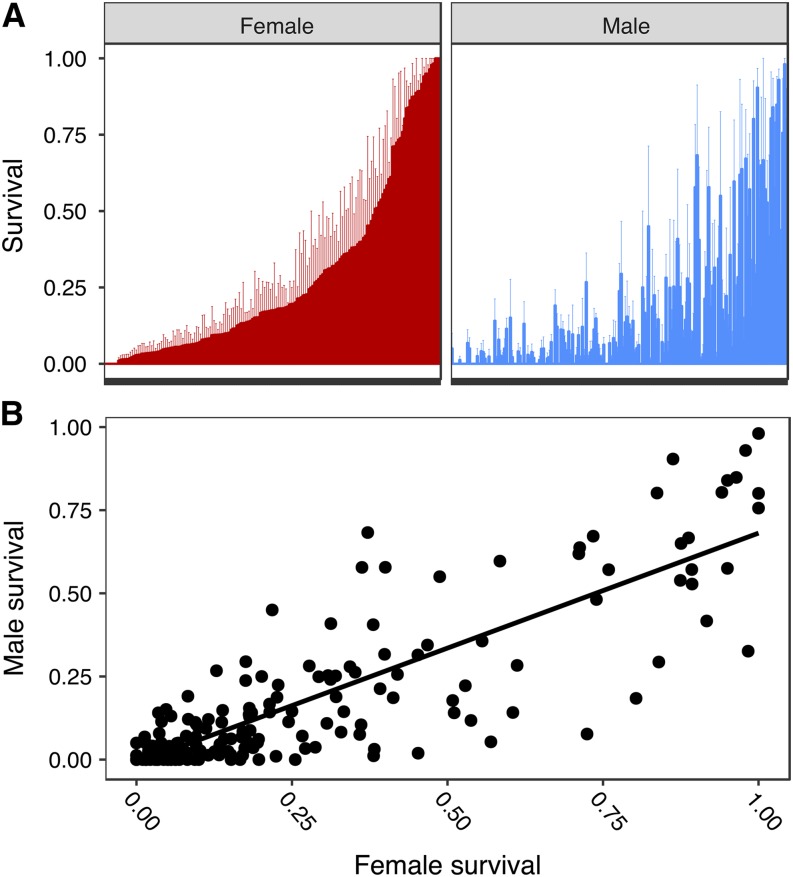
Adult survival phenotype showing A.) mean survival ± SE for each line after 6 h of anoxic exposure. Survival is sorted by female survival. B) Male and female survival was strongly correlated (*r* = 0.839, *P* < 0.0001).

Survival phenotypes were collected for male and female adults in 171 lines after exposure to 6 hr of anoxia (Figure 1A, File S1). Survival ranged from 0–100% in females and 0–98% in males. There was a significant amount of genetic variation in anoxia tolerance, with a broad-sense heritability (*H^2^*) of 0.552 (Table 1). Adults exhibited a sexually dimorphic response to anoxic exposure with females being generally more tolerant than males (main effect of sex, *P* < 0.0001, Table 1). However, a nonsignificant interaction term (Table 1) suggests that although females are more tolerant, the genotype did not affect the difference between males and females. A strong phenotypic correlation (Figure 1B) suggests that lines with high anoxia tolerance in females generally meant higher tolerance in males, while a cross genetic correlation of *r_gs_* = 0.999 indicates that the genetic architecture to anoxia survival is nearly identical between sexes. Together these suggest that most polymorphisms identified by the GWA should affect both sexes, yet not without the possibility of some sex-specific polymorphisms.

**Table 1:** Analysis of Variance (ANOVA) for anoxia tolerance in adults and larvae. H^2^ is the broad- sense heritability

Analysis	Source of Variation	DF	MSE	*F*	*p*	*Variance (SE)*	*H^2^*
Sexes pooled	Sex	1	2.8752	62.652	*<*0.0001	fixed	0.552
	Line	169	0.4572	9.962	*<*0.0001	0.0545 (0.018)	
	Sex:Line	170	0.0449	0.979	0.561	0.0039 (0.0048)	
	Error	953	0.0459			0.0461 (0.007)	
Female	Line	170	0.2980	6.102	*<*0.0001	0.0681 (0.02)	0.582
	Error	479	0.0488			0.0490 (0.0101)	
Male	Line	170	0.2041	4.756	*<*0.0001	0.0437 (0.016)	0.504
	Error	474	0.0429			0.0430 (0.0095)	
Larvae	Line	168	0.0677	3.304	*<*0.0001	0.0160 (0.0099)	0.433
	Error	344	0.0204			0.0209 (0.0077)	
Adult and Larvae pooled	Stage	1	92.860	2225.410	*<*0.0001	fixed	0.475
	Line	192	0.341	8.170	<0.0001	0.0074 (0.0062)	
	Stage:Line	146	0.162	3.884	<0.0001	0.0299 (0.0143)	
	Error	1468				0.0413 (0.0053)	

**Figure 2:**
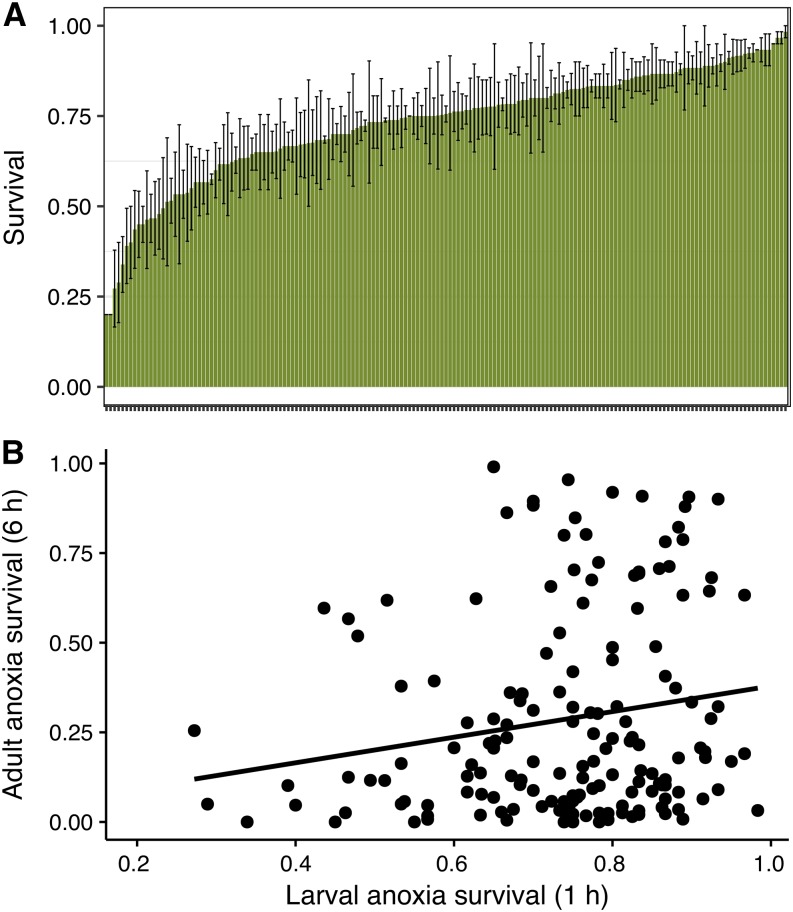
Larval anoxia phenotype showing A) mean survival ± SE for each line after 1 h of anoxic exposure. B) Larval survival was significantly, but not strongly correlated to adult survival pooled by sex (*r* = 0.18, *P* = 0.02).

For larvae, survival was measured for 169 lines after exposure to 1 hr of anoxia (Figure 2A, File S1). Larval survival varied tremendously between lines and spanned a range of 20–98% survival with a broad sense heritability of *H^2^*= 0.433 (Table 1). Escape behavior measured as the proportion of larvae climbing out of the media during anoxic exposure also varied greatly, ranging from 13.3–88.3%; climbing was only marginally significant with a low correlation (*r* = 0.16, *P* = 0.047, Figure S1)). Anoxia tolerance in larvae was correlated with combined-sex adult anoxia tolerance (Figure 2B). However, the low phenotypic correlation between adult and larval anoxia tolerance (*r* = 0.18, *P* = 0.02, Figure 2B), indicates that lines with high larval tolerance may not have high tolerance as adults. The low cross-genetic correlation (*r_gs_* = 0.249) suggests that, in general, the genetic architecture affecting anoxia tolerance may indeed be substantially different between larvae and adults.

Additionally, we correlated our anoxia phenotypes with numerous other physiological and life history traits previously measured using the DGRP to better understand the genetic correlation between anoxia tolerance and other phenotypic traits measured in the DGRP (Table S1). Correlations were conducted on 16 traits in adults pooled by sex, 52 in males, 51 in females, and 35 traits in larvae; this includes the most current phenotypes measured using the DGRP and our criteria for selection of phenotypes was limited to those whose sex (male, female, or pooled) and stage (larvae or adult) was given. Anoxia tolerances in both males and females were positively correlated with three traits [chill coma recovery (Males: *r* = 0.207, *P* = 0.006; Females: *r* = 0.160, *P* = 0.016), starvation resistance (Males: *r* = 0.156, *P* = 0.029; Females: *r* = 0.143, *P* = 0.022; Mackay *et al.* 2012), and alcohol tolerance (Morozova *et al.* 2015)] and negatively correlated with one trait [sensitivity to oxidative stress (Males: *r*=-0.157, *P* = 0.035; Females: *r*=-0.135, *P* = 0.037; Jordan *et al.* 2012)]. Of the 8 traits found to be significantly correlated with anoxia-tolerance in only males (Table S1), anoxia tolerance was most strongly correlated with decreasing susceptibility to unfolded proteins (*r*=-0.317, *P* = 0.001; Chow *et al.* 2013). Of the 11 traits found to be significantly correlated with anoxia tolerance in only females, anoxia was most correlated with resistance to fungal infection (*r* = 0.424, *P* = 0.001; Wang *et al.* 2017) and decreasing lipid content (*r*=-0.343, *P* = 0.001; Nelson *et al.* 2016). For larvae, 6 traits were correlated with anoxia tolerance (Table S1). The most correlated trait was State 3 mitochondrial respiration rate (*r* = 0.415, *P* = 0.011; Jumbo-Lucioni *et al.* 2012). Additionally, larval anoxia tolerance was positively correlated with two traits for resistance to oxidative stress (Jordan *et al.* 2012; Weber *et al.* 2012).

### Genome-wide association analyses of anoxia tolerance

GWA analyses were performed for mean anoxia tolerance in adults and larvae exposed to 6 h and 1 h of anoxia, respectively. Analyses were performed on five traits: the anoxia-tolerance of pooled males and females, the difference in anoxia-tolerance between sexes (female anoxia tolerance – male anoxia tolerance), the anoxia-tolerance of males and females separately, and the anoxia-tolerance of larvae. Line means had an approximate normal distribution (Quantile-Quantile plots shown in Figure S2 and S3). Prior to analyses, data were tested for the effect of *Wolbachia* infection, polymorphic inversions, and the amount of polymorphic relatedness (Huang *et al.* 2014). There was a significant effect of the inversion *In_2R_NS* on anoxia-tolerance in males, females, and the difference between sexes—9 SNPs and 5 candidate genes identified were found within this inversion (Table S2). Additionally, there was a significant effect of the inversion *In_2L_t* on the difference between sexes in anoxia-tolerance—18 SNPs and 12 candidate genes were located within this inversion (Table S2). There was no effect of Wolbachia infection on anoxia tolerance.

Using a nominal threshold of *P* < 10^−5^, the GWA for adults found 180 SNPs associated with the ability to survive 6 h of anoxic exposure (Figure S4A, File S2); of these SNPs, 119 genes were identified from 147 SNPs located in or within 1 kb. Of the SNPs located within 1 kb from a known gene, 51.4% were found in intronic regions and the remaining SNPs fell within coding regions (∼4.0%), untranslated regions (UTR, 5.0%) and intergenic regions (35.1%). For larvae, the GWA found 32 SNPs in or near 22 genes to be significant (Figure S4B, File S3); the majority of SNPs were found in intronic regions (55.3%), 13.1% fell within coding regions, while the remainder fell within UTR (7.9%) and intergenic (21.1%) regions. Interestingly, only three genes overlapped between life stages (Female & Larvae: *Eip63F-1*, Adult Difference & Larvae: *mamo*, Female & Adult Average & Larvae: *CG42266*; Figure S5).

Gene ontology enrichment analyses were conducted to group genes with related function to investigate possible physiological mechanisms responsible for anoxia tolerance. Adult genes clustered within 12 functional clusters; of the four significant clusters with an Enrichment Score > 2, two were related to ion transport (Table S3). Gene ontology enrichment for larval genes returned two enriched groups that involve alternative splicing and the SH3 domain (Table S4).

### Functional testing of candidate genes

**Figure 3:**
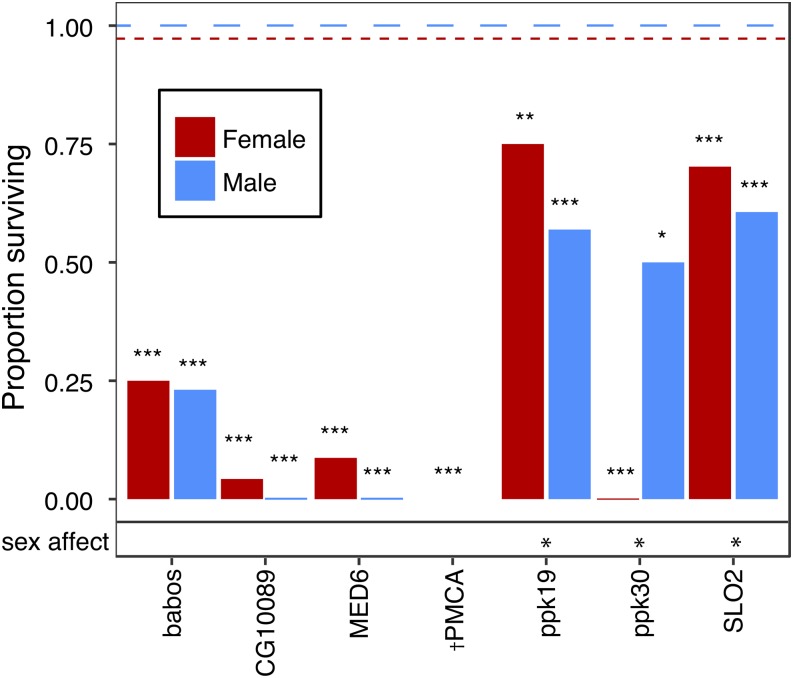
Results of the RNAi-based functional experiments for seven genes identified in the GWA for adults exposed to 6 h of anoxia. Likelihood ratio tests were used to compare each RNAi knockdown to the control for each sex. The dashed lines represent the survival proportions for control groups exposed to anoxia for each sex. All genes significantly reduced anoxia tolerance using RNAi-mediated knockdown. * *P* < 0.05, **P < 0.01, ***P < 0.001. †only females were assayed.

**Figure 4:**
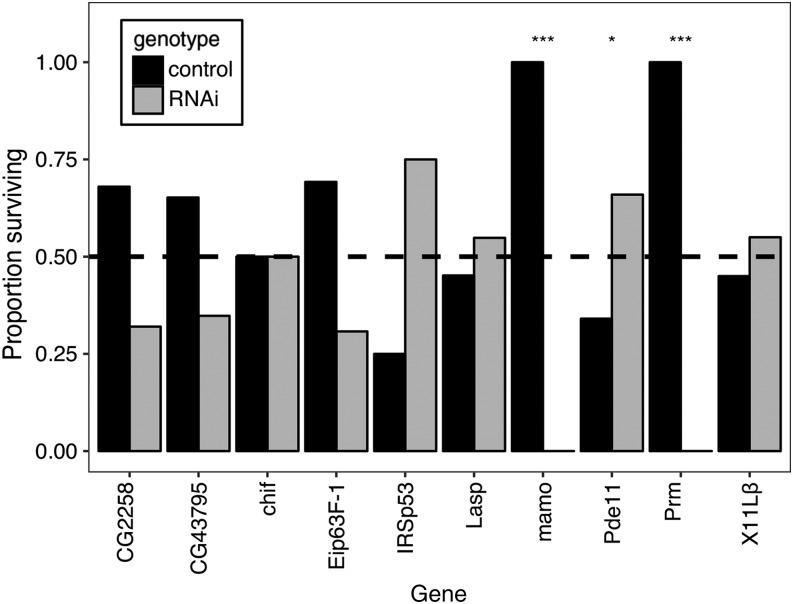
Results of the RNAi-based functional experiments for ten genes identified by the GWA for larvae exposed to 1 h of anoxia. Values are shown as the proportion of each genotype surviving 1 h of anoxic exposure. The dashed line represents the expected ratio if there was no effect of the knockdown on surviving anoxia. Likelihood ratio tests were used to compare RNAi knockdown to the control. * *P* < 0.05, **P < 0.01, ***P < 0.001

We were able to functionally test seven genes identified in the GWA for adult anoxia tolerance and ten genes for larval anoxia tolerance using RNAi-mediated knockdown. Genes were chosen based on RNAi line availability and their involvement processes linked to anoxia tolerance. The *ubiquitous-GAL4* driver line did lead to strong lethality in some lines which limited the number of genes we could experimentally test for adults; additionally, only adult females were tested in the RNAi knockdown of *PMCA* (plasma membrane Ca^++^ ATPase) because of male lethality. For adult anoxia tolerance, all RNAi knockdown animals had significantly lower anoxia tolerance relative to controls (Figure 3, Table S5). Only three of the knockdowns showed differences between sexes (Figure 3, Table S6). While some of the effects were moderate, other RNAi knockdowns led to very low survival. The genes *babos*, *CG10089*, *MED6* (Mediator complex subunit 6), and *PMCA* had the most significant effects overall and were all below 25% survival. The effects of the RNAi knockdowns were not hugely sex-specific with the exception of *ppk30* (pickpocket 30), in which 50% of males survived and no females survived anoxic exposure; the mapped allelic effects from the GWA for the SNP associated with *ppk30* were also sex-specific with females predicted to be more tolerant. For larval anoxia tolerance, only three of the ten genes selected for functional experiments were significantly different from controls (*P* < 0.05, Figure 4, Table S7). Unlike in adults, the direction of the effects of the RNAi knockdowns in larvae varied. Of the three genes that influenced survival, RNAi knockdowns of the genes *mamo* (maternal gene required for meiosis) and *Prm* (paramyosin) had lower survival than controls, while RNAi of *Pde11* (phosphodiesterase 11) had higher survival than controls.

**Figure 5:**
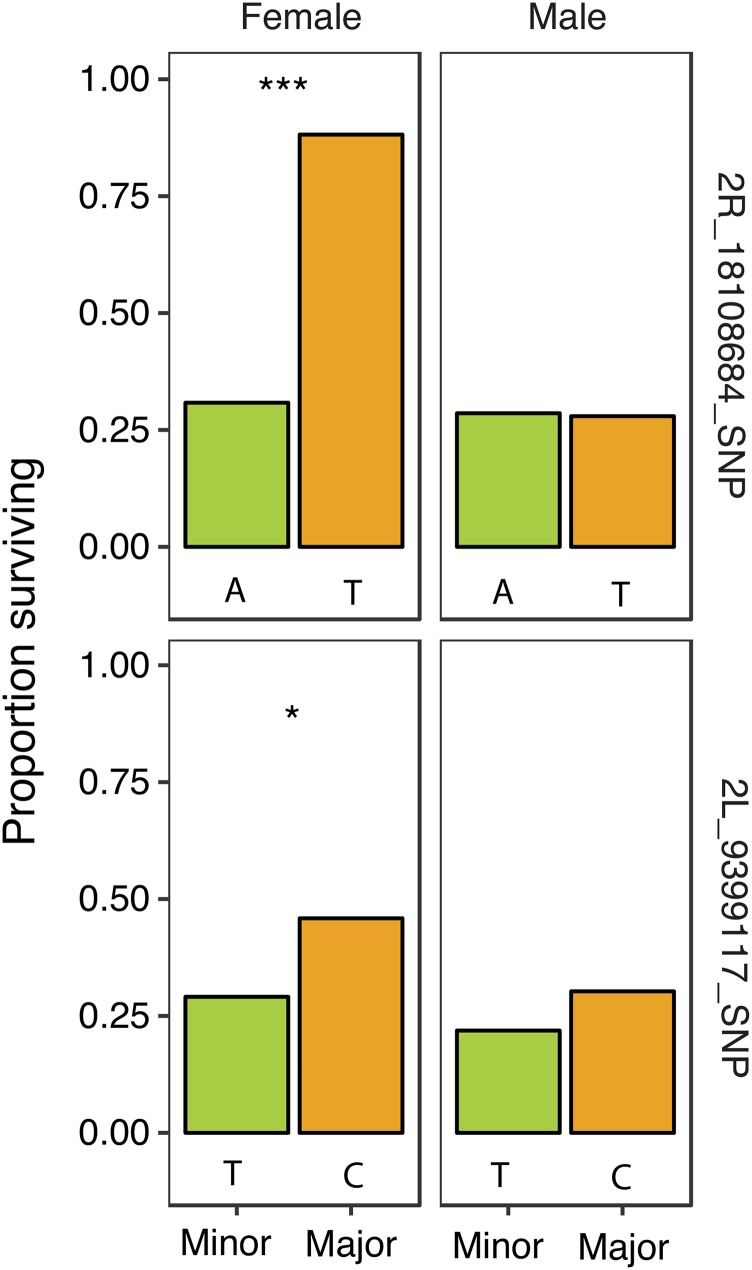
Results of the SNP-based functional experiments for two SNPs associated with adult anoxia tolerance. Each row represents the identified SNP. Likelihood ratio tests were used to compare the two alleles separately for each sex. * *P* < 0.05, **P < 0.01, ***P < 0.001

We also selected two SNPs and tested the effect of SNP allele on anoxia tolerance, using a method described by Garlapow *et al.* (2015). By crossing randomly selected lines with either the major or minor allele, we created ten F_1_ genotypes that were homozygous for either the major or the minor allele with randomized genetic backgrounds elsewhere. SNPs were chosen based on a gene ontology related to ion transport. *2L*_9399117_SNP is a T/C polymorphism located in an intron of *Shawl* (Shaw-like), which encodes a voltage-gated potassium channel. *2R*_18108684_SNP is a T/A polymorphism 1 bp downstream of *Oatp58Db* (organic anion transporting polypeptide 58Db) and 291 bp upstream of *Oatp58Da* (organic anion transporting polypeptide 58Da). The SNPs were secondarily selected for differing in the strength of the sex-specific effect. In the original mapping, lines with the minor allele were more anoxia tolerant in the GWAS for both SNPs chosen. For *2L*_9399117_SNP, DGRP lines with the minor allele had significantly higher anoxia tolerance in males (effect size: -0.114, *P =* 6.32 × 10^−6^, File S2) but not females (effect size: -0.110, *P* = 6.92 × 10^−4^, File S2), while *2R*_18108684_SNP, DGRP lines with the minor allele were more anoxia tolerant in females (effect size: -0.147, *P =* 7.97 × 10^−6^, File S2) but not males (effect size: -0.070, *P* = 9.48 × 10^−3^, File S2). SNP-based experiments had mixed effects on anoxia tolerance in comparison to the GWA prediction. For *2L*_9399117_SNP, the sex-specific effect was opposite of the prediction, as was the allele effect. There was no effect on anoxia tolerance between male alleles but there was an effect between female alleles; however, this effect is opposite of the GWA prediction as the major allele was more anoxia tolerant than the minor allele (Figure 5). For *2R*_18108684_SNP, the sex effect was as predicted by the GWA with significant effects of allele in females but not males; yet, the direction of the effect was opposite of the GWA prediction, as nearly 90% of females with the major allele of *2R*_18108684_SNP survived 6 h of anoxia while only 30% with the minor allele survived (Figure 5).

## Discussion

### Natural variation and heritability of anoxia tolerance

Anoxia tolerance was highly variable across DGRP lines, sexes and between life stages. Although anoxia tolerance between sexes was strongly correlated (Figure 1B), differences between sexes varied substantially among lines. In general, females were more tolerant than males, similar to that found in another fruit fly species, *Anastrepha suspense* (López-Martínez and Hahn 2012). The largest difference was within DGRP_373 where 98% of females and 32% males survived 6 h of anoxia and the smallest difference was within multiple lines (DGRP_85, DGRP_100, DGRP_105, DGRP_287, DGRP_531), wherein no females or males survived anoxic exposure. Anoxia tolerance of life stages was not strongly correlated (Figure 2B) and variation within lines was highly variable; for example, 98% of larvae from DGRP_530 survived 1 h of anoxia while only 3% of adults from the same line survived 6 h of anoxia. Together these results suggest that tolerance of anoxia depends on different genetic architecture in larvae and adults.

The broad-sense heritability of anoxia tolerance was relatively high, ranging from 0.433 - 0.582. Compared to other DGRP studies, tolerance to toxins [lead tolerance, *H^2^* = 0.76-0.80, methylmercury tolerance, *H^2^* = 0.8, (Montgomery *et al.* 2014; Zhou *et al.* 2016)] tend to have very high heritability. Tolerance to radiation, which is linked to oxidative stress, also has a very high heritability (*H^2^=* 0.8; Vaisnav *et al.* 2014). However, two other studies of oxidative stress resistance found lower heritability [acute oxidative stress *H^2^=*0.36-0.48 (Weber *et al.* 2012); chronic oxidative stress *H^2^=*0.14-0.41 (Jordan *et al.* 2012)]. The *H^2^* for anoxia-tolerance was in the range or higher than reported for other parameters related to physiological stress responses [chill coma recovery *H^2^* = 0.36 (Mackay *et al.* 2012), alcohol sensitivity *H^2^* = 0.38-0.42 (Morozova *et al.* 2015), food intake *H^2^* = 0.45 (Garlapow *et al.* 2015), mitochondrial function *H^2^* = 0.15-0.20 (Jumbo-Lucioni *et al.* 2012), resistance to fungal infection *H^2^* = 0.23-0.47 (Wang *et al.* 2017), starvation resistance *H^2^* = 0.54 (Mackay *et al.* 2012)].

### SNP-based functional experiments

We functionally tested two SNPs identified by the GWA in which the minor allele was associated with higher anoxia tolerance in adult *D. melanogaster* by creating lines that differed in these alleles within an otherwise homogenous genetic background. *2R*_18108684_SNP is located near two closely related genes—downstream of *Oatp58Db* and upstream of *Oatp58Da*—could in theory affect either gene. However, it most likely affects the closest of the two, *Oatp58Db*. Both genes play a role in organic anion transport and Malpighian tubule function (Torrie *et al.* 2004), which may help alleviate the ionic and osmotic stress associated with anoxic exposure. If that is indeed the case and if differences in SNP alleles influences anoxia tolerance, we would expect lines with the minor allele to have higher survival in anoxia; furthermore, the GWA results predicted stronger effects of the alleles in females. As predicted by the GWA, we saw higher survival for females but not males, providing a validation of the sex-specific effects of *2R*_18108684_SNP (Figure 5). *2L*_9399117_SNP is located in an intron of a gene encoding a voltage-gated potassium channel, *Shawl*. While it is located in an intronic region and may not be altering the encoded protein, it is possible that this SNP could affect gene expression. Minor alleles of this SNP were predicted by the GWA to have higher survival, with stronger effects in males. In this case, flies with the major allele had higher survival but the sex effect was the opposite to that predicted by the GWA. Females with the major allele but not males had statistically significantly higher survival (Figure 5). Thus, the directional and sex-specific effect of the alleles of *2L*_9399117_SNP on anoxia tolerance was opposite of the prediction. Despite SNP effects opposite of the GWA prediction, it may not be unusual as quantitative traits in *D. melanogaster* have been demonstrated to have complex genetic architectures involving many pleiotropic genes and alleles, wherein different alleles can vary in direction and sex-specific effects on quantitative traits (Mackay 2009).

### Correlations with immune-related responses of DGRP suggest a possible key role of immune function in anoxia tolerance

Hypoxic/anoxic bouts have been shown to illicit an inflammatory responses that can lead to organ dysfunction (Eltzschig and Carmeliet 2011; Eltzschig and Eckle 2011), suggesting that variation in the inflammatory response to anoxia might be important in explaining variation in anoxia-tolerance. Severe hypoxia induces immune genes in *D. melanogaster* (Zhou *et al.* 2007). Anoxia-tolerance in adult DGRP lines was correlated with several indices of immune function in other GWA studies. Variation in anoxia tolerance across the DGRP lines was strongly positively correlated with a resistance to fungal infection); this was the strongest correlation of all adult comparisons (Table 1, *r* = 0.424, *P* = 0.001). Further supporting some common genetic components to tolerance to anoxia and fungal infection, two genes found to be related to anoxia tolerance in our study (*sick* (sickie) and *beat-IIa*) were found to be related to resistance to a fungal infection by Wang *et al.* (2017). Anoxia-tolerance was also negatively correlated with resistance to enteric infection (Bou Sleiman *et al.* 2015; *r*=-0.291, *P* = 0.001; note the correlation here is negative because this study quantified susceptibility to infection, the inverse of resistance). Additionally, ten genes differentially expressed during an enteric infection were found in our GWA of anoxia tolerance (Bou Sleiman *et al.* 2015). Of these genes, *PGRP-LA* is a peptidoglycan recognition protein and is upregulated fourfold during an enteric infection (Bou Sleiman *et al.* 2015). These results suggest that lines with a high capacity to combat infection are more likely to survive anoxia. Compared to other inflammation-inducing phenotypes (*i.e.*, oxidative stress, cold exposure), a number of genes associated with inflammation overlap between these phenotypes and the anoxia tolerance phenotype. For example, immune response genes were identified as contributing to variation in oxidative stress resistance phenotypes (Jordan *et al.* 2012; Weber *et al.* 2012; Durham *et al.* 2014) and are shown to be upregulated in response to cold (MacMillan *et al.* 2016).

In our adult GWA, immunoglobulin-like domain genes were the most highly significant annotation gene cluster group, with eight different genes being significantly related to anoxia-tolerance. The genes within this cluster can function in immune responses and might provide a genetic mechanism for the associations between anoxia-tolerance and immune function in the DGRP lines. However, many of the specific genes, within “immunoglobulin annotation cluster 1”, that linked highly significantly with adult anoxia-tolerance have been shown to regulate neural development rather than having demonstrated immune functions (Derheimer *et al.* 2004; Evans *et al.* 2009; Farca-Luna and Sprecher 2013; Crocker *et al.* 2016), and to have moderate to high expression in the adult brain, heart and eye (Chintapalli *et al.* 2007; Celniker *et al.* 2009). Thus, these genes are more likely to affect anoxia-tolerance by non-immune-related pathways such as by affecting brain structure during development, or by influencing neuronal growth during recovery and repair of anoxia-damaged neural tissues.

### Evidence for genes mediating relationships between metabolism and anoxia tolerance

In response to anoxia, metabolism is suppressed to nearly 3% of normal (Callier *et al.* 2015). Therefore, we predicted that genes associated with metabolic processes might be important in mediating the inter-populational differences. However, the GWA study of adults did not identify any significant genes related to metabolism, suggesting that variation in metabolic genes was not particularly important in explaining variation in anoxia-tolerance across the lines. Conversely, larval anoxia tolerance is positively correlated with higher State 3 mitochondrial respiration rate across the DGRP lines (Table S1). Given that mitochondrial oxidative phosphorylation is absent in anoxia, the positive correlation between State 3 respiration rate and anoxia tolerance of larvae might arise from benefits of ATP generation during early exposure to anoxia, before the mitochondria are completely anoxic. Alternatively, mitochondria with high State 3 respiration rates might be strongly coupled, and generate less ROS during reoxygenation, or be better able to oxidatively produce ATP after sustaining damage during anoxia and reoxygenation.

Adult anoxia-tolerance was negatively correlated with glycerol and lipid content across the DGRP lines (Table S1). Lines that accumulate glycerol and lipid might be less poised for carbohydrate metabolism, which is predominant during anaerobiosis. Alternatively, high lipid contents might cause tissues to be more susceptible to damage induced by protein aggregates formed during anoxia, or ROS. Elevated cellular lipids have often been shown to repress inflammation via the PPAR pathway (Bensinger and Tontonoz 2008).

According to classical theories of anoxia-tolerance in vertebrates (Hochachka *et al.* 1996), we might have expected anoxia-tolerance to be higher in lines that accumulate higher carbohydrate stores. However, this was not the case. Our anoxia tolerance phenotype was not correlated with any studies measuring carbohydrates across the DGRP (Table S1); more specifically, there were no relationships between anoxia tolerance and glucose levels in males (Unckless *et al.* 2015) or females (Nelson *et al.* 2016). This result supports the prior finding that carbohydrate stores are not significantly depleted during anoxic exposure in *D. melanogaster* (Campbell *et al.* 2019).

### Evidence for genes that may affect anoxia-tolerance by influencing ionic homeostasis

In *D. melanogaster* exposed to anoxia, ATP depletes quickly and ionic homeostasis is lost within 30 min (Campbell *et al.* 2018), therefore we would expect that genes related to ion balance would be identified by the GWA. Gene ontology enrichment of GWA analyses for adult anoxia tolerance found twelve genes within two of the four top clusters of genes were related to ion transport/balance (Table S3, annotation clusters 3-4). RNAi-mediated knockdown of four of these genes reduced anoxia tolerance, indicating that disruption of these genes can negatively affect survival of anoxia. We tested for differences in the alleles of two SNPs identified by the GWAS as important for anoxia tolerance using a SNP-based approach and found that anoxia tolerance was indeed affected by SNP allele. Overall, these results strongly support the hypothesis that genetic variation in ion transport processes affects anoxia tolerance.

One specific class of ion transport proteins potentially related to anoxia tolerance are sodium and potassium channels. Allelic variation in the pickpocket genes (*ppk19*, *ppk30*) are associated with anoxia tolerance; these are non-voltage gated amiloride-sensitive sodium channels. RNAi-mediated knockdown of both *ppk19* and *ppk30* led to a reduction in survival after anoxic exposure (Figure 3); interestingly, males were affected by the knockdown of *ppk19* while females were affected by the knockdown of *ppk30*. Eleven different SNPs identified by the GWA were located within the gene *SLO2* (slowpoke 2), likely due to linkage disequilibrium; however, given the role of *SLO2* in ion balance, the numerous SNPs identified within *SLO2* may prove to be importance in anoxia tolerance. *SLO2* belongs to a group of high conductance and voltage-gated potassium channels that are modulated by intracellular ions (McCormack 2003); more specifically, *SLO2* channels encode Na^+^-dependent K^+^ channels that may aid neuronal excitability and the regulation of action potentials during hypoxic exposure (Budelli *et al.* 2016). We did see a significant effect of RNAi-mediated knockdown of *SLO2* on anoxia tolerance in both males and females (Figure 3), which supports other findings that suggest the ability to modulate neuronal excitability and hyperpolarization may aid in surviving anoxia (Krishnan *et al.* 1997; Ma and Haddad 1997; Ma *et al.* 2001). Also associated with anoxia-tolerance were the voltage-gated potassium channels encoded by *Elk* (Eag-like K^+^ channel) and *Shawl*. Plausibly allelic variation in these genes may affect ionic disruption during anoxia. SNP-based functional tests of *2L*_9399117_SNP (located in the intron of *Shawl*) showed significant effects on anoxia tolerance; females with the major allele were more tolerant to anoxia (Figure 5), indicating that variation in these SNPs can likely affect ion channel function in a manner that influences anoxia tolerance.

Several genes identified as linked to anoxia-tolerance are related to calcium regulation. *dpr3* (defective proboscis extension response 3) is a component of the calcium release-activated channels (CRAC channel; Vig *et al.* 2006). As calcium entry after ionic disruption is considered a key step in cell death resulting from anoxia, it is plausible that allelic variation in *dpr3* is affecting the magnitude of the calcium response to anoxia. *PMCA* was identified as a gene associated with anoxia-tolerance in adults, within the ion transport cluster. *PMCA* is a plasma membrane calcium ATPase that plays an important role in restoring intracellular calcium levels after a calcium spike (Desai and Lnenicka 2011). While the RNAi-mediated knockdown of *PMCA* only allowed for females to be tested, anoxic exposure killed all individuals with the loss of *PMCA* (Figure 3). This suggests a likely strong dependence on calcium regulation either during anoxia or the subsequent reoxygenation.

Another group of genes identified by the GWA are associated with organic anion transport. *Oatp58Da*, *Oatp58Db* and *Oatp58Dc* are organic anion transport polypeptides, identified by GWA to be associated with adult anoxia-tolerance. While all three have similar functions and are highly expressed in the Malpighian tubules (Chintapalli *et al.* 2007), *Oatp58Db* and *Oatp58Dc* have been shown to be involved in the transport of a variety of organic anions, including toxins in the Malpighian tubules (Torrie *et al.* 2004) and the blood brain barrier (Seabrooke and O’Donnell 2013; Groen *et al.* 2017). However, it is plausible that this group of genes may have functions previously unrecognized as important to anoxia tolerance. Our results show that variation at the SNP level of *Oatp58Db* (SNP) can have effects on anoxia survival; females were more tolerant to anoxia if they possessed the major allele (Figure 5). The trade-offs associated with the different allelic forms raise interesting questions; it seems plausible that one allele might be more functionally advantageous during normoxia, with the other allele providing survival advantages during stresses such as anoxia.

### Evidence for genes that affect anoxia-tolerance by affecting the resistance to oxidative stress or unfolded proteins

In order to recover from anoxia, animals must return to oxygenated air, and this reperfusion of oxygen can elicit substantial oxidative stress, which is believed by some to be the major mechanism of cell damage associated with anoxia (Ambrosio *et al.* 1987; Hashimoto *et al.* 2003; Milton *et al.* 2007). An excessive production of ROS that overcomes the buffering capacity of antioxidant defenses can lead to multiple deleterious effects on cells and organelles (Galli and Richards 2014; Pamenter 2014). For example, elevated ROS levels can oxidize unsaturated fatty acids in membranes (Behn *et al.* 2007), oxidize side chains of proteins to produce carbonyls, and oxidize nucleic acids, particularly guanine (Cooke *et al.* 2003; Dickinson and Chang 2011). Flies exposed to severe hypoxia/anoxia upregulate genes linked to antioxidant production, supporting an important role for resisting oxidative stress in anoxia-survival (Liu *et al.* 2006; Azad *et al.* 2009). If variation in anoxia tolerance involves the ability to tolerate oxidative stress, we would predict that DGRP lines more tolerant to oxidative stress would also be more tolerant of anoxia. Indeed, anoxia tolerance was significantly correlated with multiple oxidative stress-resistance phenotypes (Table S1). DGRP lines with higher anoxia tolerance were more tolerant to chronic and acute oxidative stress in both larvae and adults (Table S1). Further supporting the oxidative stress/anoxia-tolerance relationship, female anoxia tolerance was significantly correlated with virgin female lifespan (Table S1). Animals with longer lifespan tend to be more tolerant to stressors including oxidative stress (Martin *et al.* 1996). Together, these data cautiously support the hypothesis that variation in oxidative stress resistance is a component of the variation in anoxia-tolerance across the DGRP lines. Yet, the genetic mechanisms driving differences in oxidative stress responses during anoxia are unclear. The GWA did not identify any genes traditionally linked to oxidative stress resistance, such as genes involved in detoxifying or buffering ROS. Because most of the identified SNPs were not in coding regions, plausibly, variation in responses to oxidative stress are controlled by differential regulation. Alternatively, the correlations between anoxia-tolerance and tolerance to oxidative stress may arise secondarily from the effects of immune or ionic homeostatic functions that help flies cope with multiple types of stress.

Anoxia is characterized by a decrease in intracellular pH and elevations in ionic concentrations and osmotic pressures that can induce protein unfolding (Krivoruchko and Storey 2013). As a result, anoxia can cause the formation of unfolded and misfolded proteins that have exposed hydrophobic segments, rendering them prone to aggregation and degradation. Protein aggregates can be toxic to the cell, and to prevent such aggregations, cells induce molecular chaperones in response to their formation (Giffard *et al.* 2004). Large increases in protein-aggregations coincide with the increase in mortality in anoxia (Chen *et al.* 2002). Heat shock proteins (Hsps) are a group of important molecular chaperones that repair and/or protect unfolded proteins in response to numerous environmental stressors (Feder and Hofmann 1999). *Drosophila* exposed to hypoxia exhibit increased levels of Hsp70 and Hsp23, and flies with overexpressed Hsps had substantial increases in survival (Azad *et al.* 2009). Furthermore, the response of Hsps increases upon the return to normoxia (Michaud *et al.* 2011). The anoxia tolerance phenotype was strongly correlated with endoplasmic reticulum stress (Table S1), in which flies were exposed to a commonly used chemical (tunicamycin) to induce an unfolded protein response; the negative correlation reflects a measure of the inverse of survival and can be interpreted as those lines less susceptible to protein unfolding were more tolerant to anoxia. However, the genetic bases to the response to unfolded proteins in anoxia tolerance is unclear. No Hsps or other chaperones were identified by the GWA. Plausibly, upstream transcriptional regulators of Hsps might be responsible for this correlation, or the correlation between anoxia tolerance and the endoplasmic stress phenotype might be downstream of other genetic effects such as those influencing immune function or ion channels that mediate variation in stress-resistance of the DGRP lines.

### Genes related to larval anoxia-tolerance

Of the genes with known functions shown to be associated with larval anoxia-tolerance by GWA, two were DNA regulatory genes, and two were genes involved in contractile processes. *Chiffon (chif)* is in the zinc-finger superfamily and is believed to be involved in the regulation of transcription and DNA replication (Landis and Tower 1999). The gene *dre4* is a component of the FACT complex, which is involved in DNA replication and repair, and nucleosome organization (Tsunaka *et al.* 2009). Although RNAi suppression of *chif* didn’t affect anoxia tolerance (Figure 4), the GWA results suggest that alleles of *chif* and *dre4* genes could differentially influence DNA transcriptional or repair processes during or after anoxia. *Lasp* is a member of the nebulin family, and is involved in physiological processes requiring the cytoskeleton such as spermatogenesis (Lee *et al.* 2008). *Paramyosin* (*Prm*) is a muscle protein, that can modulate flight muscle stiffness, but also is expressed in larvae. RNAi suppression of *Prm* reduced anoxia tolerance, whereas knockdown of *Lasp* had no effect (Figure 4). Possibly alleles of *Lasp* and *Prm* affect locomotory behavior in the larvae, or plausibly repair processes requiring the cytoskeleton.

Another gene identified by the GWA as affecting larval anoxia tolerance is *Pde11*, which encodes a phosphodiesterase likely affecting larval locomotory behavior (Xiao and Robertson 2017). Phosphodiesterases regulate the level of cAMP and cGMP and are widely expressed in *Drosophila* tissues (Day *et al.* 2005). cGMP has been shown to modulate larval escape behavior in hypoxia (Vermehren-Schmaedick *et al.* 2010), and increased cGMP leads to a fast onset of an anoxic coma through a cGMP-activated protein kinase (Dawson-Scully *et al.* 2010). Furthermore, members of the phosphodiesterase (*Pde*) gene family mediate fast recovery from anoxic exposure (Xiao and Robertson 2017). The GWA analysis for larval anoxia tolerance identified *Pde11* as one of the top genes associated with anoxia tolerance (File S3), and RNAi-mediated knockdown of *Pde11* led to an increased larval survival (Figure 4). If *Pde11* affects anoxia tolerance by influencing locomotor behavior, we would predict that DGRP lines with larvae that exhibit less movement during anoxia would be more tolerant, as less locomotion should be correlated with reduced production of anaerobic endproducts. However, the proportion of larvae climbing out of the media when exposed to anoxia was only weakly correlated with survival (Figure S1). These results suggest that *Pde11*/cGMP phosphodiesterase activity may be regulating other processes that affect anoxia-tolerance; supporting this hypothesis, the cGMP-dependent protein kinase has been found to affect multiple physiological and behavioral parameters including dispersal activity, sleep, starvation tolerance and recovery from electroconvulsive shock in *Drosophila* (Donlea *et al.* 2012; Kelly *et al.* 2018).
